# Abstracts from Foreign Journals

**Published:** 1901-07

**Authors:** M. P. Ravenel, S. J. J. Harger

**Affiliations:** Philadelphia, Pa.; Philadelphia, Pa.


### ABSTRACTS FROM FOREIGN JOURNALS.

### FRENCH.

UNDER THE DIRECTION OF M. P. RAVENEL, M.D., AND S. J. J. HARGER, V.M.D., OF PHILADELPHIA, PA.

IODIDE OF POTASSIUM IN PARTURIENT APOPLEXY. After milking out the udder and washing it with a solution of lysol, De Bruin injects into the four quarters 10 grammes of the iodide dissolved in one litre of boiled water at a temperature of 40° C. He also recommends the injection of air, as this helps to liberate the iodine and increases the action of the drug. The cow is placed on the right side and is not milked until eighteen hours after the injection. Hypodermic administration of caffeine is recommended for cardiac weakness. The routine precautions are also taken, such as the sternal position, catheterization, and not too frequent milking.

With the old treatment 40 to 60 per cent. recovered. According to the statistics of Jensen, 1446 cases out of 1744 recovered with the iodide treatment—83 per cent.; 2.5 per cent. died from pneumonia. Danish records show that in 1144 cases 73 per cent. were able to get up eighteen hours, and 53 per cent. twelve hours, after the first injection. In many cases in which the iodide is not effective it is a question of mistaken diagnosis.—*Annales de Méd. Vét.*

TREATMENT OF PLEURISY IN THE HORSE WITH THORACENTESIS AND SALINE INJECTIONS. Brocherion recently treated successfully eight cases of pleurisy with repeated thoracentesis and the subcutaneous injection of a sodium chloride solution of 1 : 1000.

Almy effected two rapid recoveries with the same treatment in addition to the usual medicinal preparations ; the salt solution was injected into the jugular vein. Concerning the last two cases, in the first horse thoracentesis was practised twice, and thirteen litres of the salt solution were given in seven injections. In the second horse the thorax was punctured four times, and twenty litres of salt solution were injected. In both cases the recovery was rapid, and the intravenous treatment seemed to have had a beneficial influence upon the disease. Being accustomed to a mortality of 70 to 80 per cent. in this disease, the author views with much encouragement future trials with this treatment. The chest is aspirated whenever the pleural effusion is sufficiently abundant to cause distressed breathing. The salt solution is administered at the same time and also between the intervals of aspiration if the symptoms become alarming. From two to three litres are given at a time.

Brocherion, who administers the solution hypodermically, selects the region posterior to the shoulder. The needle is attached to a stationary vessel so regulated that the solution flows at the rate of a litre per hour.

Butel considers the mortality of simple cases of pleurisy diagnosed early only about 20 per cent. Very acute pleurisy with abundant exudation of blood is nearly always fatal in a short time. Pleurisy complicating pneumonia of strangles is excessively grave. Those which complicate epizoötics of infectious pneumonia as well as simple acute pleurisy are not so fatal. Puncture of the chest when practised antiseptically is harmless, and should not be neglected in order to make an early diagnosis. —*Rec. de Méd. Vét.*

Laceration of the Retina. This lesion was noticed by Schimmel in a horse, aged twelve years. The eye was normal excepting two white spots on the cornea and a cloudiness of the lens and vitreous humor. The ophthalmoscope revealed a transverse laceration of the retina in the posterior part of the eyeball and a little below the centre. The retina below the rent was thickened and had the appearance of a clear, blue sky studded with small white fleecy clouds. The seat of the laceration was covered with small white points resembling cholesterin crystals; the bottom of the eye was a yellowish aspect and the optic papilla was very indistinctly visible. Vision was lost, and no treatment was advisable.—*Annales de Méd. Vét.*

Etiology and Treatment of Periodic Ophthalmia. Dr. Dor, a Lyons oculist, claims to have discovered the microbe of periodic ophthalmia, and recommends iodide of potash in large doses. By inoculating a culture of this microbe into the vitreous humor of the eye he succeeded in producing in the rabbit and in the horse a recurring ophthalmia similar to that in the horse. Its periodicity he explains thus: The microbe thrives better in a neutral and acid rather than in an alkaline medium. The media of the eye, especially the vitreous humor, have an acid reaction, and hence the localization of the microbe in the eye. The white blood-corpuscles during the inflammatory process, through their secretion, render the ocular media alkaline, and hence the arrest of the disease. The microbes which have not been destroyed by phagocytosis will resume the pathological process when the media have again assumed their normal acidity. The author concludes that the treatment should aim to render the media alkaline. Alkaline preparations, such as bicarbonate of sodium, and even caustic soda itself, have not been successful, and the better results obtained from iodide of potash can scarcely be explained in this manner.

In horses inoculated with the microbe the author succeeded in preventing the development of the diseased process, while in the check animal the eye became diseased. The dose of the iodide employed was 15 to 18 grammes daily intravenously, and 25 to 30 grammes by the mouth. In one case treated a large hypopion was absorbed in forty-eight hours. The daily dose suggested is 25 to 30 grammes.

The author also observes that on soils which produce forage containing an abundance of lime and other diverse alkaline elements the disease is rare, while on a low, marshy ground, where the forages are deficient in these salts, periodic ophthalmia is enzoötic.

He also mentions the circumstance that many veterinarians have succeeded in arresting such an epizoötic by feeding the animals on forage obtained from limestone soil. The experiments will be continued. —*Rec. de Méd. Vét.*

Therapeutic Application of Light. Garnault recalls the fact that workmen affected with rheumatism are completely cured by being exposed for forty-eight hours to an intense electric light; also that in manufactories employing electricity for soldering, which is accompanied with very intense light, the workmen are cured of rheumatism and gout.

The author concludes from his experiments with this agent as follows: Hot or cold light can be used with advantage in a number of affections. Among these are chronic articular and muscular rheumatism, varicose ulcers, anginas, tonsillitis, chronic nasal catarrh, ozæna, chronic auricular catarrh accompanied with buzzing, and deafness.—*Rec. de Méd. Vét.*

Pulmonary Antisepsis in Strangles Infection. The general therapeutics of strangles, according to the essay of Pruneau here reviewed, includes more especially antisepsis of the respiratory tract, and hence the administration of drugs by means of tracheal injections.

The respiratory mucous membrane has a high absorbing power, demonstrated by Colin :

1. A horse received during six hours twenty-five litres of water into the lungs through a tracheal incision. Every two hours six kilos (thirteen pounds) of blood were abstracted.

2. A horse was killed in ten minutes by a tracheal injection of 12 grammes of alcoholic extract of nux vomica dissolved in 250 grammes of water.

3 Colin found in the blood taken from the jugular vein cyanide of iron and potash four minutes after 50 grammes of this salt had been injected into the trachea.

When made slowly intratracheal injections are very well tolerated by the horse and the rabbit, and for this reason it is a safe, economic, and sure means of medication recommended by a number of veterinarians. Levi, of the Veterinary School of Pisa, says: The intratracheal administration is suitable for most drugs soluble in ether or alcohol. Only the oily and too irritating should not be given in this manner. Beside, to avoid coughing and bronchitis, the solution should be well diluted and injected slowly.

Nevertheless, Levi has given oily tracheal injections, and recommends the following formula in gangrenous pneumonia :

Olive oil and essence of turpentine, each 5 to 15 grammes, according to the gravity of the disease.

Twenty c.c. of the following formula have been given with impunity :

℞.—Olive oil, 20 grammes ; iodoform, 2 grammes ; essence of turpentine, 10 grammes.—M.

The following agents have been studied :

1. *Iodoform.* Iodoform has a powerful influence upon the microbes of putrefaction. Under the combined influence of oil, heat, bacteria, and ptomaïnes it is slowly decomposed and liberates the iodine, which is an antiseptic and so much more powerful as it is in its nascent state.

2. *Essence of Turpentine.* This is very efficacious against chronic bronchial catarrh, and rapidly destroys the fetid odor of the expectoration. Given in the formula of Levi, it does not provoke coughing.

3. *Creosote.* It is a powerful antiseptic but too irritating to the mucous membrane, and is not to be recommended.

4. *Eucalyptol.* It is very soluble in oil, and is three times more bactericidal than phenic acid. Under its influence the cough diminishes; the expectoration becomes less abundant, less purulent, and more fluid. In gangrenous pneumonia it acts like essence of turpentine. Locally it is stimulant and antiseptic to the mucous membranes. It is not irritating, even in the proportion of one part of eucalyptol to two of oil.

5. *Guaiacol.* This agent is not irritating. It lessens the cough and renders the expectoration more fluid and less purulent. Pignol recommends the following formula: ℞.—Eucalyptol, 14 grammes; guaiacol, 5 grammes; iodoform, 1 gramme; sterilized olive oil, q. s. 1 c.c. For one injection given from three to twelve times daily.

6. *Thymol.* Very soluble in oils. Pure, it is very irritating to the mucous membranes, but may be substituted by the essence of thyme.

7. *Oil of Gaultheria.* This is a salicylate of methyl, of antiseptic value, superior to salicylic acid and equal to phenol; not irritating.

8. *Menthol.* Diluted 1 : 20 it is not irritating. It is strongly antiseptic, and has beside analgesic properties almost equal to cocaine.

The author recommends the following polypharmaceutic formula, of which 20 c.c. are injected: ℞.—Eucalyptol, 5 grammes; guaiacol, 5 grammes; menthol, 5 grammes; essence of thyme, 10 grammes; essence of wintergreen, 10 grammes; essence of conella, 10 grammes; essence of turpentine, 30 grammes; iodoform, 10 grammes; olive oil, 150 grammes.

Mode of Action. The effects are mechanical and chemical. The oil lessens the mucous secretions and renders expectoration more easy and less painful. The discharge is, therefore, more abundant during the first few days following the treatment if the catarrhal inflammation extends to the lungs.

*Chemical Action.* 1. Disinfection of the bronchi and lung alveoli during inspiration, and of the trachea, larynx, pharynx, and nasal cavities during expiration by the evaporation of the essences and the liberation of the iodine.

2. Drying of the mucous membranes. The moist râles disappear quite rapidly.

3. Cessation of cough and discharge.

4. Finally, by the absorption of the medicinal substances by the pulmonary mucous membrane the temperature can be lowered and the organism strengthened by the return of the appetite.

To make the injection, the hypodermic syringe is employed, the needle being inserted between the tracheal rings. The injection is made at the rate of 20 c.c. per minute.

With this treatment the author enumerates the details of nine cases of unilateral and bilateral strangles pneumonia treated successfully. After the tracheal injections were commenced all other medication ceased. Usually two doses are given daily. There seems to have been a prompt amelioration of the symptoms.—*Rec. de Méd. Vét.*

